# Multi-sensory Responsiveness and Personality Traits Predict Daily Pain Sensitivity

**DOI:** 10.3389/fnint.2019.00077

**Published:** 2020-01-10

**Authors:** Tami Bar-Shalita, Sharon A. Cermak

**Affiliations:** ^1^Department of Occupational Therapy, School of Health Professions, Sackler Faculty of Medicine, Tel Aviv University, Tel Aviv, Israel; ^2^Chan Division of Occupational Science and Occupational Therapy at the Herman Ostrow School of Dentistry, University of Southern California, Los Angeles, CA, United States; ^3^Department of Pediatrics, USC Keck School of Medicine, Los Angeles, CA, United States

**Keywords:** sensory over responsiveness, sensory modulation, pain sensitivity, pain perception, risk factor, personality traits

## Abstract

**Background**: A continuous effort has been devoted to identifying factors that contribute to individual differences in pain perception. Amongst the personality traits, Neuroticism is assumed to be the most significant moderator of experimental and clinical pain. Multi-sensory responsiveness to daily sensations has been shown to be associated with pain perception. Yet, neither the relationship between personality traits and multi-sensory responsiveness nor the impact of both these factors to pain perception have been examined. Thus, this study aims to explore the contribution of both multi-sensory responsiveness and personality traits to pain perception in a daily context.

**Methods**: A community-based sample of 204 adults completed the Sensory Responsiveness Questionnaire-Intensity Scale (SRQ-IS); the Big Five Inventory (BFI); and the Pain Sensitivity Questionnaire (PSQ).

**Results**: The partial eta-square demonstrated that the *SRQ-IS Aversive* sub-scale score had the strongest relationship with the PSQ-Total score, accounting for 9% of the variation. The regression coefficient relating PSQ-Total score with *SRQ-IS Aversive*, and BFI sub-scales of *Extraversion, Neuroticism and Openness-to-Experience* scores was found to be *r* = 0.39 (*p* < 0.0001), accounting for 16% of the variance, and yielding a large effect size.

**Discussion**: To the best of our knowledge this is the first study to report on the interplay between aversive responsiveness to daily sensations and personality traits of *Neuroticism, Openness-to-Experience, and Extraversion* as contributing factors to daily pain sensitivity, amongst which aversive responsiveness was found as the major contributing factor. This study may broaden the understanding of the pain experience variability, both in practice and in experimental research.

## Introduction

Pain is a compound multifaceted experience composed of sensory, affective, and cognitive processes (Moayedi and Davis, 2013). There is substantial individual variability in the perception of experimental and clinical pain, as well as in the susceptibility in developing painful conditions, and responding to pain-relieving treatments (Mogil, 1999; Pud et al., 2004, 2006). Continuous efforts have been devoted to identifying factors relevant to understanding this variability (Pud et al., 2004, 2014; Vassend et al., 2013). Increasing evidence indicates that genetic factors (Young et al., 2012; Vassend et al., 2013), demographic characteristics (e.g., age, sex, ethnicity) and personality traits (Riley and Wade, 2004; Pud et al., 2004)—the prompts to think or act in a similar way in response to varied stimuli or situations (Goldberg, 1990), are all related to pain responses. Further, an ecological perspective to painful events in life situations posits that pain is not isolated, and maybe experienced more intensely in individuals who are over-responsive to stimuli derived from other sensory modalities (Bar-Shalita et al., 2015, 2019).

Sensory modulation affects the ability to grade responses to stimuli across one or more sensory systems (ICDL, 2005; Miller et al., 2007); Sensory over-responsivity (SOR) manifests as a condition in which non-painful stimuli are perceived as abnormally irritating, unpleasant (ICDL, 2005; Miller et al., 2007) or painful (Bar-Shalita et al., 2012, 2014; Weissman-Fogel et al., 2018) consequently interfering with participation in daily life (Dunn, 2007; Bar-Shalita et al., 2008; Chien et al., 2016), and in quality of life (Kinnealey et al., 2011; Bar-Shalita et al., 2015). Testing the association between sensory responsiveness and daily pain perception indicated that increased daily pain sensitivity co-occurs with SOR (Bar-Shalita et al., 2015). Furthermore, experimental pain findings suggest atypical pain processing and modulation in subjects with SOR demonstrated by pain hypersensitivity (Bar-Shalita et al., 2014; Weissman-Fogel et al., 2018). Interestingly, while pain hypersensitivity is also related to personality traits (Pud et al., 2004), personality traits are impacted by sensory processing (Dunn, 2001; Croy et al., 2011).

The five-factor model of personality dimensions (Goldberg, 1990) includes (1) *Agreeableness—*being sympathetic, kind, and affectionate; (2) *Conscientiousness—*being organized, thorough, and reliable; (3) *Extraversion—*being talkative, energetic, and assertive; (4) *Openness to experience—*having wide interests and being imaginative and insightful; and (5) *Neuroticism—*being tense, moody, and anxious. The personality trait of Neuroticism is considered to be among the most significant moderators of experimental and clinical pain (Wade and Price, 2000; Boggero et al., 2014). Since, individuals with SOR demonstrate enhanced experimental pain ratings, as well as daily pain hypersensitivity (Bar-Shalita et al., 2012, 2014, 2015), we hypothesized that Neuroticism together with SOR will best explain the variance of daily pain sensitivity than either of these factors alone. Importantly, the five-factor model presents traits that are clearly dimensional (Chaplin et al., 1988), thus personality can be best understood by assessing the ranks on these five bipolar factors (McCrae and John, 1992). Yet, neither the importance of Neuroticism nor the association of the other personality traits with pain responses have been sufficiently studied. Of note, since the presence of pre-existing pain may alter the perception of pain sensation (Apkarian et al., 2011; Woolf, 2011), or influence the self-reporting of personality traits (Fishbain et al., 2006), and since we aimed at contributing to a better understanding of the pre-existing individual factors that may impact pain perception, this study investigated a non-clinical, healthy sample.

## Materials and Methods

This is a cross-sectional study, approved by the institutional ethics review committee, and all participants provided written consent before enrolling in the study.

### Participants

The participant population has been included in a previous publication, authored by both authors of this article (Bar-Shalita and Cermak, 2016). A non-clinical convenience sample of 204 adults [51.5% (*N* = 105) men] participated in this study. Mean (SD) age was 27.4 (3.71) years (age range 23–40 years). The study sample included 48.5% of university students, while the rest (51.5%) were recruited off-campus and reported work as their main occupation. Eighty-five percent were native-born while the rest (15%; *n* = 30) were born in Europe, the USA, and Africa. Forty-seven percent had up to 12 years of education, while 53% had higher education. As for family status 76% were single and the rest were married. Exclusion criteria stipulated pregnancy, frequent or chronic pain conditions, neurodevelopmental conditions including autism and ADHD, neurological deficits including speech, vision, hearing or behavioral abnormalities, a history of psychopathology as well as any restrictions to self-reporting.

### Instrumentation

#### The Sensory Responsiveness Questionnaire-Intensity Scale (SRQ-IS; Bar-Shalita et al., 2009a)

A self-report questionnaire assessing responses to daily sensations, aiming at clinically identifying sensory modulation dysfunction. The scale consists of a set of 58 items that represent typical scenarios encountered occasionally throughout daily life. Each scenario involves one sensory stimulus in one modality including auditory, visual, gustatory, olfactory, vestibular and somatosensory stimuli excluding pain. The items are worded in a manner that attributes a hedonic/aversive valence to the situation [e.g., Aversive sample item: *It bothers me the way new clothes feel*; Hedonic sample item: *I enjoy loud noises (such as a vacuum cleaner, construction work)*]. The participant rates the intensity of the hedonic/aversive response to the situation using a 5-point scale with the anchors “*not at all”* attached to the score of “1” and *“very much”* attached to the score of “5.” Two scores are computed: sensory responsiveness questionnaire (SRQ)-Aversive (32 items) assessing SOR and SRQ-Hedonic (26 items) assessing sensory under-responsivity (Mean SD 1.87 **+** 0.26; 2.10 **+** 0.33, respectively). The SRQ has been demonstrated to have content, criterion and construct validity, as well as internal consistency (Cronbach’s *α* = 0.90–0.93) and test-retest reliability (*r* = 0.71- 0.84; *p* < 0.001–0.005; Bar-Shalita et al., 2009a).

#### The Big Five Inventory (BFI; John et al., 1991)

A 44-item self-report questionnaire assessing five broadband personality traits: *Extraversion*, encompassing such traits such as talkative, energetic, and assertive; *Agreeableness*, being sympathetic, kind, and affectionate; *Conscientiousness*, being organized, thorough, and reliable; *Neuroticism*, being tense, moody, and anxious; and *Openness to experience*, having wide interests and being imaginative and insightful. The response format utilizes a 5-point Likert scale varying from “*total disagreement*” attached to the score of “1” to “*total agreement*” attached to the score of “5.” A sum score for each of the five personality dimensions is used to build a personality profile. The Big Five Inventory (BFI) questionnaire has been demonstrated to have content, convergent and discriminant validity, as well as internal consistency (Cronbach’s *α* = 0.79–0.87; Mean 0.83; Worrell and Cross, 2004; John et al., 2008).

#### The Pain Sensitivity Questionnaire (PSQ; Ruscheweyh et al., 2009)

A 17-item self-report questionnaire assessing daily pain sensitivity based on pain intensity ratings of imagined painful daily life situations in different somatosensory sub-modalities. The Pain Sensitivity Questionnaire (PSQ) is suggested as an alternative to experimental pain procedures that evaluate pain sensitivity in healthy and chronic pain patients (Ruscheweyh et al., 2009, 2012). Pain intensity is rated on a scale with the anchors “*not painful at all”* attached to the score of “0” and *“worst pain imaginable”* attached to the score of “10.” The PSQ provides a total score (*PSQ-total*) and two subscale scores: *PSQ-moderate* (sample item: *Imagine you burn your tongue on a very hot drink*) and *PSQ-minor* (sample item: *Imagine you prick your finger tip on the thorn of a rose*). The PSQ has been demonstrated to have content, criterion and construct validity, as well as internal consistency (Cronbach’s *α* = 0.92 for *PSQ-total*, 0.81 for *PSQ-minor* and 0.91 for *PSQ-moderate*), and test-retest reliability (ICCs = 0.83, 0.86 and 0.79, respectively; Ruscheweyh et al., 2009).

### Procedure

A convenience sample of participants, recruited using a snowball sampling, were contacted by phone. Information regarding the study was provided by the researcher while inclusion criteria were verified. Eligible participants attended a session, where after completing a consent form and a medical and demographic questionnaire, they were administered the SRQ, BFI, and PSQ. The latter three questionnaires were completed on a counter-balanced order to avoid sequential effects and to balance the possible influence of fatigue and attention span. The session lasted for approximately 45 min, with the researcher present and available for participants’ queries.

### Data Analysis

Statistical analyses were performed with SAS V9.3 (SAS Institute, Cary, NC, USA). Continuous variables are summarized with a mean and standard deviation and categorical variables are presented by a count and percentage. Pearson’s correlation coefficient is presented between pairs of continuous variables with a level of significance. Linear regression was performed to assess multiple correlation coefficients (R) regression coefficients and effect sizes (partial eta-square) with 95% confidence limits presented. All statistical tests were two-sided and tested at a 5% level of significance. Since this was an exploratory study with no existing previous data relating to SRQ and BFI, adjustments for multiple testing were not performed and nominal *p*-values are presented.

## Results

Descriptive statistics (Mean; SD) for the three study measures (BFI; SRQ; PSQ) is presented in Table 1.

**Table 1:** Descriptive statistics (Mean; SD, Min, Median and Max) for the three study measures (BFI; SRQ; PSQ; *N* = 204).

Measures		Mean	SD	Min	Median	Max
BFI	Extraversion	3.4	0.69	1.7	3.4	4.9
	Neuroticism	2.7	0.71	1.0	2.6	4.6
	Agreeableness	3.8	0.56	1.9	3.8	5.0
	Conscientiousness	3.8	0.60	2.2	3.8	5.0
	Openness-to-Experience	3.6	0.53	2.0	3.7	4.9
SRQ	Aversive	1.9	0.32	1.3	1.9	2.8
	Hedonic	2.1	0.32	1.3	2.2	2.9
PSQ	Total	61.9	22.31	0.0	63.0	117.0
	Moderate	22.0	9.29	0.0	22.0	49.0
	Minor	36.0	11.53	0.0	37.0	64.0

*Note. BFI, The Big Five Inventory assessing personality traits; SRQ, The Sensory Responsiveness Questionnaire-Intensity Scale assessing sensory responsiveness subtypes; PSQ, The Pain Sensitivity Questionnaire*.

### Association Between Personality Traits (BFI) and Sensory Responsiveness (SRQ)

Low to moderate statistically significant correlations were found between SRQ scores and BFI scores in the total sample (*n* = 204; Table 2). The SRQ-Aversive score showed significant correlations with all BFI scores except Openness-to-Experience. The SRQ-Hedonic score correlated significantly with two of the five BFI scores; such that a negative weak correlation was found with the Neuroticism score, whereas a weak positive correlation was found with the Openness-to-Experience score (Table 2).

**Table 2:** Pearson correlation coefficients between the SRQ (Aversive and Hedonic) scores and the BFI (Extraversion, Neuroticism, Agreeableness, Conscientiousness, Openness to Experience) scores (*N* = 204).

BFI	SRQ	
	Aversive	Hedonic
Extraversion	−0.26***	0.09
Neuroticism	0.39***	−0.16*
Agreeableness	−0.21**	0.00
Conscientiousness	−0.17*	−0.10
Openness-to-Experience	−0.05	0.28***

Note. SRQ, The Sensory Responsiveness Questionnaire-Intensity Scale assessing sensory responsiveness subtypes; BFI, The Big Five Inventory testing personality traits. *p < 0.05, **p < 0.01, ***p < 0.001.

### Association Between Personality Traits (BFI), Sensory Responsiveness (SRQ) and Daily Pain Sensitivity (PSQ)

The total, moderate and minor scores on the PSQ were found to have statistically significant low correlations with the SRQ-Aversive score but not with the SRQ-Hedonic score. Furthermore, the PSQ scores were found to have statistically significant low positive correlations with Neuroticism and negative correlations with Openness-to-Experience (Table 3).

**Table 3:** Pearson correlation coefficients between the SRQ (Aversive and Hedonic), the BFI (Extraversion, Neuroticism, Agreeableness, Conscientiousness, Openness to Experience) and the PSQ (Total, Moderate, Minor and Non-painful) scores (*N* = 204).

Sensory measure (SRQ)	PSQ tot	PSQ mod	PSQ min
SRQ—Aversive	0.29***	0.24***	0.32***
SRQ—Hedonic	0.04	0.05	0.016
**Personality measure (BFI)**
Extraversion	0.05	0.08	0.00
Neuroticism	0.29***	0.20**	0.23***
Agreeableness	−0.13	−0.11	−0.12
Conscientiousness	−0.07	−0.06	−0.05
Openness to Experience	−0.15*	−0.14*	−0.15*

Note. SRQ, The Sensory Responsiveness Questionnaire-Intensity Scale assessing sensory responsiveness subtypes; BFI, The Big Five Inventory assessing personality traits; PSQ, The Pain Sensitivity Questionnaire: tot-total; mod-moderate; min-minor sub-scales. *p < 0.05, **p < 0.01, ***p < 0.001.

### Assessing Contributing Factors to Pain Perception

In order to assess the contributions of both sensory and personality factors to pain perception, all variables that were significantly correlated with the PSQ scores were entered into multivariate model. These variables also were found either to be correlated with the PSQ scores or with the SRQ scores in the univariate analyses (Tables s 2, 3).

The PSQ-Total score was significantly correlated with the SRQ-Aversive score and the Extraversion, Neuroticism, and Openness to Experience scores of the BFI (Table 4; multivariate correlation coefficient *r* = 0.39, *p* < 0.0001).

**Table 4:** Regression coefficients relating the PSQ-Total score with the SRQ-Aversive, and BFI-Extraversion, Neuroticism, and Openness-to-Experience scores (*R*^2^ = 0.156).

Parameter	Estimate	Standard error	*t*-Value	*p*-Value
Intercept	15.32	16.27	0.94	0.3474
SRQ-Aversive	18.94	5.03	3.77	0.0002
Extraversion	6.57	2.27	2.89	0.0042
Neuroticism	5.50	2.26	2.43	0.0161
Openness	−7.41	2.81	−2.63	0.0092

*Note. PSQ, The Pain Sensitivity Questionnaire; SRQ, The Sensory Responsiveness Questionnaire-Intensity Scale assessing sensory responsiveness subtypes; BFI, The Big Five Inventory assessing personality traits*.

The partial eta-square demonstrated that the SRQ-Aversive score has the strongest relationship with the PSQ-Total score, accounting for about 9% of the variance which is considered a medium effect size. The personality components of Extraversion, Neuroticism and Openness-to-Experience each contributed about 2% to the total variation (Table 5). SRQ-Aversive, Extraversion, Neuroticism and Openness-to-Experience together, as noted above, account for 16% of the variation, i.e., the model has a large effect size (Cohen, 1988). The resulting linear equation is as follows:

**Table 5:** The contribution of each parameter (SRQ-Aversive and BFI: Extraversion, Neuroticism and Openness-to-Experience scores to the variation of the PSQ-Total score (Partial eta-square with 95% confidence limits).

	Partial eta-square	95% Confidence limits	
SRQ-Aversive	0.0937	0.0302	0.1733
Extraversion	0.0203	0.0000	0.0727
Neuroticism	0.0259	0.0002	0.0821
Openness	0.0336	0.0020	0.0942

*Note. SRQ, The Sensory Responsiveness Questionnaire-Intensity Scale assessing sensory responsiveness subtypes; BFI, The Big Five Inventory assessing personality traits; PSQ, The Pain Sensitivity Questionnaire*.

PSQ-Total = 15.32 + 18.94*SRQ-Aversion + 6.56*Extraversion + 5.50*Neuroticism–7.41*Openness-to-Experience. Similar patterns were found for the PSQ- Moderate and Minor scores (data not shown).

## Discussion

To the best of our knowledge, this is the first study that investigated the contribution of personality traits and multi-sensory responsiveness to the individual variance in daily pain perception in a non-clinical healthy sample, in an attempt to explore potential pre-existing individual factors that may affect pain perception. Results demonstrate that aversive responsiveness to sensations and the personality traits of Neuroticism, Openness-to-Experience and Extraversion all contribute to daily pain sensitivity. Specifically, individuals who were most sensitive to pain tended to be high in aversive responsiveness to multi-sensory stimuli and in Neuroticism while low in Openness-to-Experience, and in Extraversion, with sensory aversive responsiveness measuring SOR, was found as the major contributing factor to pain sensitivity.

### Sensory Responsiveness and Personality Traits

People vary in the way they perceive their environment (Croy et al., 2011) which contributes to the characterization of their personality traits (McCrae et al., 2000). The “sensory filter” hypothesis is based on the notion that people do not have an objective picture of the environment surrounding them, but rather a person-specific filtered one (Croy et al., 2011). Accordingly, an individual’s sensory processing capacity would partly form such a sensory-filter system that is applied when perceiving sensory events, robustly impacting the perceived world, and in turn, influencing one’s customary thoughts, emotions and behavior relative to the environment, which characterize personality traits (McCrae et al., 2000). Thus, when considering the trajectory that determines the way people perceive the environment, it seems tenable that the sensory processing ability may influence the way the world is conceived, which then develops into a pattern of behavioral responses. But at the same time, the sensory system’s capacities and personality traits may both share the same genetic origins (Croy et al., 2011). Moreover, basic behavioral characteristics may be predisposed but also are developed and shaped with accumulating experiences within the environment (Croy et al., 2011). Hence, elusive shaping of underlying genetic elements of personality are environmentally enabled, and an individual pattern of sensory responses may be related and contribute to personality characteristics (McCrae et al., 2000). Indeed, research has demonstrated significant individual variability in sensory abilities (McCrae et al., 2000), as well as in the tolerance to the pain sensory system (John et al., 1991; Fillingim et al., 2009; Paine et al., 2009).

### Personality Traits and Pain Perception

The five-factor model of personality, considered to have a biological basis (Jang et al., 2002), was designed to supply a comprehensive taxonomy of traits using five basic dimensions (Goldberg, 1990). Positive traits are as interesting and significant as the more familiar negative traits when studying the factors underlying individual variability in pain perception (Vassend et al., 2013). This study demonstrates that when examining the association between personality traits and daily pain sensitivity, the subscales of Neuroticism (positive correlation) and Openness-to-Experience (negative correlation) were found significantly associated. Pud et al. (2014) sub-grouped healthy individuals according to different pain modality sensitivities and personality profiles, and found that the personality trait of harm avoidance was the most likely to determine pain perception. Harm avoidance, according to Cloninger’s Tridimensional Personality Theory, is defined as a tendency to respond intensely to previously established signals of aversive stimuli and to passively avoid novelty (Paine et al., 2009). In the present study, Neuroticism was found to have the strongest correlations (among all five personality traits) to all three daily pain sensitivity measures. While Neuroticism is characterized by tenseness, moodiness, and anxiety (Martínez et al., 2011; Littman-Ovadia and Lavy, 2012), it is the trait most similar to harm avoidance (Pud et al., 2014). Openness-to-Experience, which this study found negatively correlated to daily pain sensitivity, denotes having wide interests and being imaginative and insightful (Littman-Ovadia and Lavy, 2012). It seems that Openness-to-Experience could serve as the opposite anchor of harm avoidance. Notably, while Pud et al. (2014) tested the relation between pain sensitivity and personality dimensions in the lab, our findings not only support their results, but have the additional advantage of being able to be extrapolated to environments outside the lab.

### Contributors to Pain Perception

This study found that the main contributor to pain likelihood was the SRQ-Aversive score, which surprisingly far exceeded the importance of personality traits. The SRQ Aversive sub-scale contains items that reflect irritation from daily non-noxious sensations. We have previously reported that individuals with over-responsiveness to daily sensation demonstrate hyperalgesia and lingering sensation to experimental pain stimuli (Bar-Shalita et al., 2009b, 2012, 2014, 2019). Indeed, individuals who are sensory over-responsive process sensory stimulus more intensely, longer and become overwhelmed by everyday sensory experiences (ICDL, 2005; Miller et al., 2007; Davies et al., 2010). Consequently, one of their main adaptive coping mechanisms reported is avoidance (Kinnealey et al., 1995). Harm avoidance, which was previously found as the principal factor that seems to determine pain perception (Pud et al., 2014), and was reported to be highly associated with Neuroticism as well (Caseras et al., 2003), leads to fear-avoidance behavior (Conrad et al., 2007), and worsens pain perception (Pud et al., 2004; Vlaeyen and Linton, 2006). Specifically, higher Harm avoidance was found correlated to less efficient endogenous analgesia, assuming to characterize pro-nociceptive individuals (Nahman-Averbuch et al., 2016). Thus, we suggest that the predisposition of aversive responsiveness to sensations can lead to avoidance. These, in turn, evolve into fear-avoidance behaviors which consequently may be demonstrated as a pro-nociceptive pain perception (Bar-Shalita et al., 2019). Using a multivariate model enabled a more authentic examination allowing a dimensional perspective of all factors tested. As such this is the first study to indicate that these three personality traits *(Extraversion, Neuroticism and Openness to Experience)* similarly contribute to pain perception. Moreover, our findings demonstrate that pain perception has a stronger link to the sensory domain than to the personality domain.

### Study Limitations

There are limitations to this study that warrant attention: this study measured daily pain perception through self-report. Although the measure used (PSQ) is suggested by its authors as an experimental pain procedures alternative for evaluating pain sensitivity in healthy and chronic pain patients (Ruscheweyh et al., 2009, 2012), objective experimentally induced pain measures were not carried out in this study. Further, though the study population varied in geographical and vocational variables, with approximately 50% of university students, this study utilized a convenience sample. Moreover, distribution in most demographic variables may indicate that these did not impact research findings, however future research should investigate personality and sensory responsiveness together with cultural, religious, previous painful experiences, and ethnicity to better capture the pain sensitivity phenomenon. Finally, though we found a large effect size, a causal relationship cannot be claimed using this study design.

## Conclusion

The presence of pain may either alter the perception of pain sensations (Apkarian et al., 2011; Woolf, 2011), or influence the self-reporting of personality traits (Fishbain et al., 2006). Thus, in order to shed light on the pre-existing individual factors that could affect pain perception, this study investigated a non-clinical, healthy sample. Findings illuminate the key role that sensory responsiveness has in daily pain sensitivity and may have an important implication in preventing pain as well as in pain therapy. Moreover, the similar contribution of Openness-to-Experience and Extraversion as Neuroticism in predicting pain highlights the complexity of pain perception. Effective pain treatment can only be achieved by approaching the entire person, rather than the biological pathology (de Meij and van Kleef, 2016). Hence, the identification of sensory responsiveness patterns and specific personality traits can together allude to the pain perception profile and contribute to an individually tailored multidisciplinary pain management therapy.

## Data Availability Statement

The raw data supporting the conclusions of this article will be made available by the authors, without undue reservation, to any qualified researcher.

## Ethics Statement

The studies involving human participants were reviewed and approved by School of Occupational Therapy, Hebrew University. The patients/participants provided their written informed consent to participate in this study.

## Author Contributions

Both authors listed have made a substantial, direct and intellectual contribution to the work, and approved it for publication.

## Conflict of Interest

The authors declare that the research was conducted in the absence of any commercial or financial relationships that could be construed as a potential conflict of interest.
